# UvrD Participation in Nucleotide Excision Repair Is Required for the Recovery of DNA Synthesis following UV-Induced Damage in *Escherichia coli*

**DOI:** 10.1155/2012/271453

**Published:** 2012-09-27

**Authors:** Kelley N. Newton, Charmain T. Courcelle, Justin Courcelle

**Affiliations:** Department of Biology, Portland State University, Portland, OR 97201, USA

## Abstract

UvrD is a DNA helicase that participates in nucleotide excision repair and several replication-associated processes, including methyl-directed mismatch repair and recombination. UvrD is capable of displacing oligonucleotides from synthetic forked DNA structures *in vitro* and is essential for viability in the absence of Rep, a helicase associated with processing replication forks. These observations have led others to propose that UvrD may promote fork regression and facilitate resetting of the replication fork following arrest. However, the molecular activity of UvrD at replication forks *in vivo* has not been directly examined. In this study, we characterized the role UvrD has in processing and restoring replication forks following arrest by UV-induced DNA damage. We show that UvrD is required for DNA synthesis to recover. However, in the absence of UvrD, the displacement and partial degradation of the nascent DNA at the arrested fork occur normally. In addition, damage-induced replication intermediates persist and accumulate in *uvrD* mutants in a manner that is similar to that observed in other nucleotide excision repair mutants. These data indicate that, following arrest by DNA damage, UvrD is not required to catalyze fork regression *in vivo* and suggest that the failure of *uvrD* mutants to restore DNA synthesis following UV-induced arrest relates to its role in nucleotide excision repair.

## 1. Introduction

 The accurate duplication of the genome is critical to the survival of any organism. DNA damage, such as that caused by UV irradiation, can disrupt the replication machinery and prevent it from completing its task [1, 2]. In *Escherichia coli*, a number of the cellular events associated with the recovery of replication forks arrested by UV-induced lesions are known to involve several gene products in the RecF pathway [3–5]. Following replication arrest, the nascent lagging stand of DNA is partially degraded through the coordinated activity of the RecJ nuclease and RecQ helicase [4]. The extent of degradation is limited by RecF-O-R, which facilitates loading and formation of a RecA filament at the stalled fork. Both biochemical and cellular studies suggest that RecF, -O, and -R, together with RecA, facilitate strand exchange or regression at the branch point of the arrested fork [6, 7]. Cellular studies suggest that this processing restores the lesion-containing region to a double-stranded form, allowing nucleotide excision repair to access and repair the lesion [6, 8]. In the absence of either processing or repair, the recovery is delayed and elevated levels of rearrangements, mutagenesis, and lethality are observed [8–10]. A number of other gene products have also been postulated to participate in aspects of the recovery process but have yet to be examined *in vivo*.

 UvrD is a DNA helicase that participates in both nucleotide excision repair and replication-associated processes. Nucleotide excision repair is the process by which bulky adducts and lesions are removed and repaired from DNA [11, 12]. During nucleotide excision repair (NER), a heterotetramer, UvrA_2_UvrB_2_, recognizes and binds the damaged region [11, 13, 14]. UvrD acts after incision to release the resulting 10 to 12 bp oligonucleotide and UvrB-UvrC complex from the DNA [15–18]. The resultant gap is then filled in by DNA polymerase I and sealed by DNA ligase [19].

 During replication, UvrD function is required to displace the nascent DNA strand during methyl-directed mismatch repair, a replication-coupled process that removes mispaired bases [20, 21]. It is required for replication of several rolling-circle plasmids [22] and copurifies with DNA polymerase III holoenzyme under some conditions [23]. Conjugational and transformational recombination frequencies increase in *uvrD *mutants [24, 25] and decrease in strains overexpressing UvrD [26]. In addition, *uvrD* mutants are constitutively induced for the SOS response and show elevated levels of RecA foci [27, 28].

 The concept that UvrD may process replication forks following arrest comes from a number of genetic observations. *uvrD *mutants exhibit synthetic lethality with *rep* [29, 30], which encodes another 3′-5′ helicase that is required for the replication of phage ΦX174 and some plasmids [31, 32] and is postulated to remove obstacles on the DNA during replication such as bound proteins or transcriptional machinery [33, 34]. Viability in *uvrD rep* double mutants can be restored by mutations in *recF, recO, *and *recR*, which are required to process and restore replication following arrest by DNA damage [5, 6, 35]. Subsequent studies found that purified UvrD was capable of displacing oligos and RecA filaments from synthetic replication fork structures *in vitro* [36, 37]. These observations led some researchers to speculate that, in addition to its other roles, UvrD function may participate in displacement of the lagging strand and RecA filament from arrested replication forks [37, 38]. However, the molecular function of UvrD at replication forks has not been directly examined *in vivo*. 

 Here we characterize the role of UvrD at the replication fork following arrest by UV-induced damage *in vivo*. We find that UvrD is necessary for DNA synthesis to resume following UV irradiation. However, the initial degradation, processing and regression of the arrested fork occur normally in the absence of UvrD. Similar to other mutants deficient in nucleotide excision repair, the regressed fork structures fail to resolve in *uvrD* mutants and continue to accumulate and persist. These observations indicate that UvrD is not required to catalyze fork regression *in vivo* and support the idea that the hypersensitivity and failure to restore replication in the absence of UvrD are likely due to its role in nucleotide excision repair. 

## 2. Materials and Methods

### 2.1. Bacterial Strains

All bacterial strains used in this study are in an SR108 background, a *thyA36 deoC2 *derivative of W3110 [39]. SR108, CL579 (SR108 *recF332*::Tn*3*), HL952 (SR108 *uvrA*::Tn*10*), HL1054 (HL108 *uvrD*::tetR), and HL944 (SR108 *recQ1803*::Tn*3*) have been described previously [4, 6, 8, 40]. CL1272 (DY320 *uvrD*::*kan*) was constructed using the recombineering strain DY329 [41]. The kanamycin resistance gene was amplified from Tn5 using PCR primers 5′CCCAACCTATTTTTACGCGGCGGTGCCAATGGACGTTTCT- ATGGACAGCAAGCGAACCG3′ and 5′AGGCCAAATAAGGTGCGCAGCACCGCATC-CGGCAACGTTATCAGAACTCGTCAAGAAG3′. The PCR product was then transformed into DY329 to generate CL1272, selecting for kanamycin resistance. The gene replacement was transferred into SR108 using standard P1 transduction to generate CL1302 (SR108 *uvrD*::*kan*). 

### 2.2. UV Survival Studies

Fresh overnight cultures were diluted 1 : 100 in DGCthy medium (Davis medium supplemented with 0.4% glucose, 0.2% casamino acids, and 10 *μ*g/mL thymine) and grown to an OD_600 _ of between 0.4 and 0.5 at 37°C in a shaking bath. Serial dilutions of each culture were plated in triplicate on Luria-Bertania plates supplemented with 10 *μ*g/mL thymine and UV irradiated with the indicated doses. A Sylvania 15 watt germicidal lamp (254 nm) delivering an incident dose of 0.9 J/m^2^/s (0.2 J/m^2^/s for doses less than 20 J/m^2^) was used for all irradiations. Plates were grown overnight at 37°C, and colonies were counted the following day.

### 2.3. Recovery of DNA Synthesis

Fresh overnight cultures were diluted 1 : 100 in 50 mL DGCthy medium supplemented with 0.1 *μ*Ci/mL [^14^C]-thymine and grown to an OD_600 _of 0.3 at 37°C in a shaking water bath. Half of each culture was mock-irradiated, and the other half was irradiated with an incident dose of 27 J/m^2^. At the indicated times, duplicate 0.5 mL aliquots of each culture were pulse-labeled with 1 *μ*Ci/mL [^3^H]-thymidine for 2 min at 37°C. The cells were then lysed and the DNA precipitated using ice-cold 5% trichloroacetic acid (TCA). The DNA was filtered onto 2.4 cm Fisherbrand glass fiber filters, and the amount of ^14^C and ^3^H was determined using a liquid scintillation counter.

### 2.4. DNA Degradation Assay

Fresh overnight cultures were diluted 1 : 100 in 6 mL DGCthy medium supplemented with 0.1 *μ*Ci/mL [^14^C]-thymine and grown to an OD_600_ of 0.3 in a shaking water bath at 37°C. At this point, cultures were pulse-labeled for 5 s with 1 *μ*Ci/mL [^3^H]-thymidine, filtered onto a 0.45 micron Millapore filter, and rinsed twice with 1X NET buffer (100 mM NaCl, 10 mM EDTA, pH 8.0, 10 mM Tris, pH 8.0). The cells were then resuspended in 10 mL nonradioactive, prewarmed DGCthy media, UV-irradiated at an incident dose of 27 J/m^2^, and incubated in a 37°C shaking water bath. Triplicate 0.2 mL samples were taken at time zero, followed by duplicate samples every 20 min for the duration of the experiment. These samples were added to 5% TCA to lyse the cells and precipitate the DNA. The DNA was filtered onto 2.4 cm Fisherbrand glass fiber filters, and the amount of radioactivity on each filter was measured using a liquid scintillation counter.

### 2.5. Two-Dimensional Agarose Gel Electrophoresis

Cultures harboring the pBR322 plasmid were grown overnight in DGCthy medium in the presence of 100 *μ*g/mL ampicillin. One milliliter of this culture was pelleted, resuspended at a 1 : 100 ratio in 20 mL of DGCthy medium, and grown without ampicillin to an OD_600_ of 0.5 at 37°C in a shaking water bath. The cultures were then UV-irradiated with 50 J/m^2^, and 0.75 mL aliquots were transferred to an equal volume of ice-cold 2X NET (200 mM NaCl, 20 mM EDTA pH 8.0, 20 mM Tris, pH 8.0) at the times indicated. These samples were then pelleted, resuspended in 0.14 mL of lysis buffer (1.5 mg/mL lysozyme, 0.5 mg/mL RNase A in 10 mM Tris, pH 8.0, 1 mM EDTA, pH 8.0), and incubated at 37°C. After 30 min, 10 *μ*L of 20% Sarkosyl and 10 *μ*L of 10 mg/mL proteinase K were added and the incubation was continued for 30 more min. The samples were then extracted twice with 4 volumes of phenol/chloroform/isoamyl alcohol (25 : 24 : 1) and extracted once with 4 volumes of chloroform/isoamyl alcohol (24 : 1). The samples were dialyzed against 200 mL of TE buffer (2 mM Tris, pH 8.0, 1 mM EDTA, pH 8.0) for 1 h on floating 37 mm Whatman 0.05 *μ*m pore discs and digested with PvuII overnight at 37°C. The samples were loaded onto 0.4% agarose gel following extraction with 2 volumes of chloroform/isoamyl alcohol (24 : 1) and run at 25 V for 16 hours in 1X TBE buffer (Tris-borate-EDTA, pH 8.0). For the second dimension, the lanes were excised, rotated 90 degrees, recast in a 1% agarose gel in 1X TBE, and the gel was electrophoresed at 200 V for 7 h. The DNA from the gels was transferred to a HybondN+ nylon membrane by standard Southern blotting, and the plasmid DNA was detected with an [*α*-^32^P]dCTP (MP Biomedicals) labeled pBR322 probe that was prepared using a nick translation protocol (Roche). Radioactivity was visualized and quantified using a Storm 840 Phosphoimager and ImageQuant software (GE LifeSciences).

## 3. Results

### 3.1. UvrD Is Required to Restore DNA Synthesis following Arrest by UV Damage

**Figure 1:**
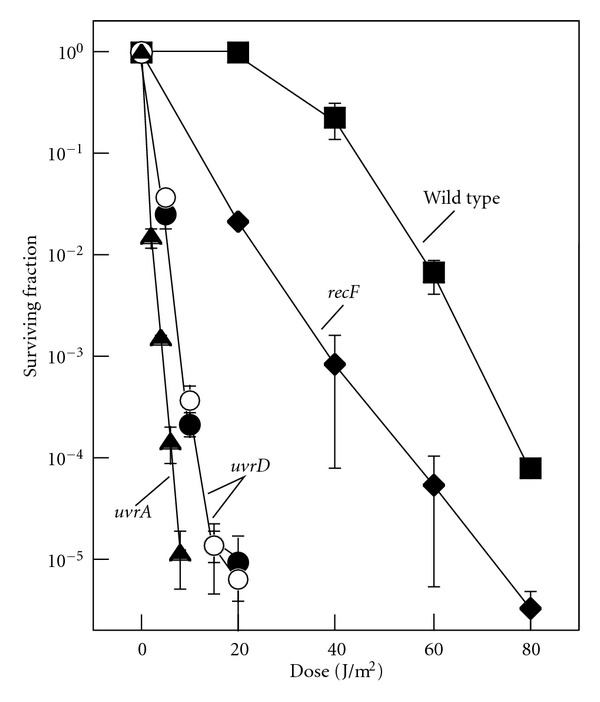
Cells lacking UvrD are hypersensitive to irradiation with UV light. Survival of wild-type (■), *recF *(◆), *uvrA *(▲), *uvrD::kan *(*⚫*), and *uvrD::tet *(◯) cultures following irradiation with the indicated UV doses. Error bars represent the standard error of the mean.

 We constructed a UvrD deletion, *uvrD::kan* and compared its UV resistance along with a previously characterized strain, *uvrD::tet *(parental strain HL 1054 [40]), with that of *uvrA *and *recF *mutants (Figure 1). UvrA is required for the initial step of nucleotide excision repair and RecF is required for processing and maintaining forks arrested by UV-induced damage. Both *uvrD *mutations rendered cells more sensitive to UV irradiation than wild-type cells. Consistent with previous studies, *uvrD *mutants are more sensitive than *recF *mutants [42] but less sensitive than *uvrA *mutants [43]. The higher resistance of *uvrD *compared with *uvrA* can be explained by its role in turnover of UvrC. *uvrD *mutants retain a limited ability to carry out nucleotide excision repair, and remain proficient in repairing 6-4 photoproducts, which are removed preferentially before cyclobutane pyrimidine dimers or lesions in transcribed genes [40]. It is also important to note that *recF *mutants are able to withstand considerably more UV exposure than *uvrD *mutants. The RecF protein is involved in the stabilization of disrupted replication forks, and, consequently, the susceptibility of *recF *mutants to UV damage is related to the frequency with which the replication machinery encounters a lesion. In the presence of nucleotide excision repair, the frequency of these events is substantially decreased. Therefore, that the *uvrD *mutant is considerably more sensitive than the *recF *mutant would indicate that the UvrD protein still plays a substantial role in nucleotide excision repair.

**Figure 2:**
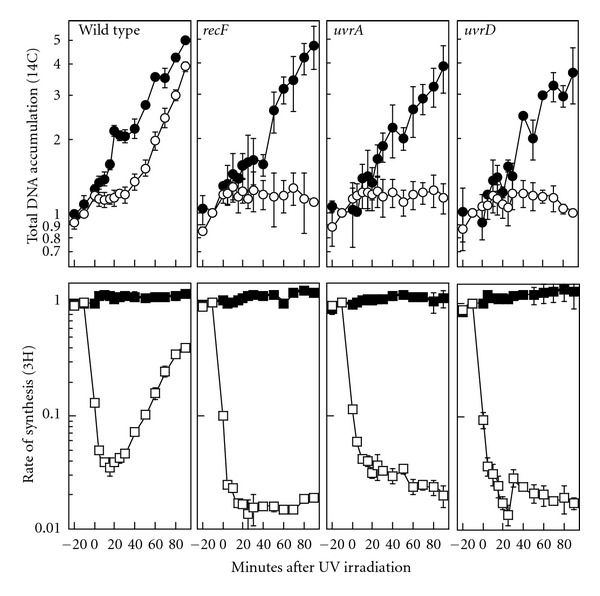
UvrD is required for the recovery of replication following UV irradiation, but not for replication in the absence of damage. Cells grown in the presence of [^14^C]-thymine were pulse-labeled for 2 min with [^3^H]-thymidine at the times indicated following either UV irradiation with 27 J/m^2^ (open symbols) or mock irradiation (closed symbols). Total DNA accumulation (^14^C incorporation, circles) and rate of synthesis (^3^H incorporation/ 2 min, squares) are plotted. Graphs represent the average of at least three independent experiments. Error bars represent the standard error of the mean. The level of [^3^H] and [^14^C] in preirradiated DNA ranged between 30,000–50,000 cpm and 3000–6000 cpm for all experiments.

 To determine if UvrD is required to resume DNA replication following arrest by UV-induced damage, we monitored DNA synthesis over time in UV-irradiated cultures of *uvrD *mutants. Cultures grown in the presence of ^14^C-thymine were UV-irradiated or mock-irradiated and allowed to recover over a period of 90 min. The rate of synthesis was monitored by pulse-labeling aliquots of the culture with ^3^H-thymidine for two min at various times during the recovery period. In this manner, both the total DNA accumulation (^14^C-incorporation) and the rate of synthesis (^3^H-incorporation/2 min) can be followed simultaneously. By this assay in wild-type cells, the rate of synthesis dropped over 90% immediately following UV-irradiation and then began to recover after approximately 20 min and approached preirradiation levels by the end of the 90 min time course. A transient pause in the accumulation of DNA in the wild-type culture was also observed consistently at times prior to when replication resumed (Figure 2). For the purposes of comparison, we also examined mutants lacking RecF and UvrA, which have been shown previously to be defective in the resumption of replication following arrest by UV damage [5]. In these mutants, no further DNA accumulation was observed following irradiation and the rate of synthesis did not recover (Figure 2). When we examined UV-irradiated cultures of *uvrD*, we observed that the rate of DNA synthesis was inhibited to a similar extent as in *recF *and *uvrA *cultures after irradiation and also failed to recover (Figure 2). Additionally, no further accumulation of DNA was observed in these cultures. The results indicate that UvrD is necessary for the resumption of replication following arrest by UV-induced damage. 

### 3.2. UvrD Does Not Contribute to the Nascent DNA Processing That Occurs following Arrest at UV-Induced Damage

**Figure 3:**
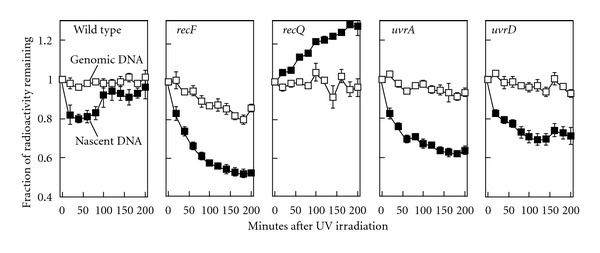
In the absence of UvrD, the nascent DNA at stalled replication forks is degraded in a manner similar to other repair mutants. [^14^C]-thymine-labeled cultures were pulse-labeled with [^3^H]-thymidine for 5 s before the cells were collected, resuspended in nonradioactive media, and UV-irradiated with 27 J/m^2^. The fraction of ^14^C-labeled genomic DNA (□) and ^3^H-labeled nascent DNA (■) remaining over time is plotted. Graphs represent the average of three independent experiments. The level of [^3^H] and [^14^C] in DNA immediately preceding irradiation ranged between 2500–7000 cpm and 1000–2500 cpm in all experiments. Error bars represent the standard error of the mean.

**Figure 4:**
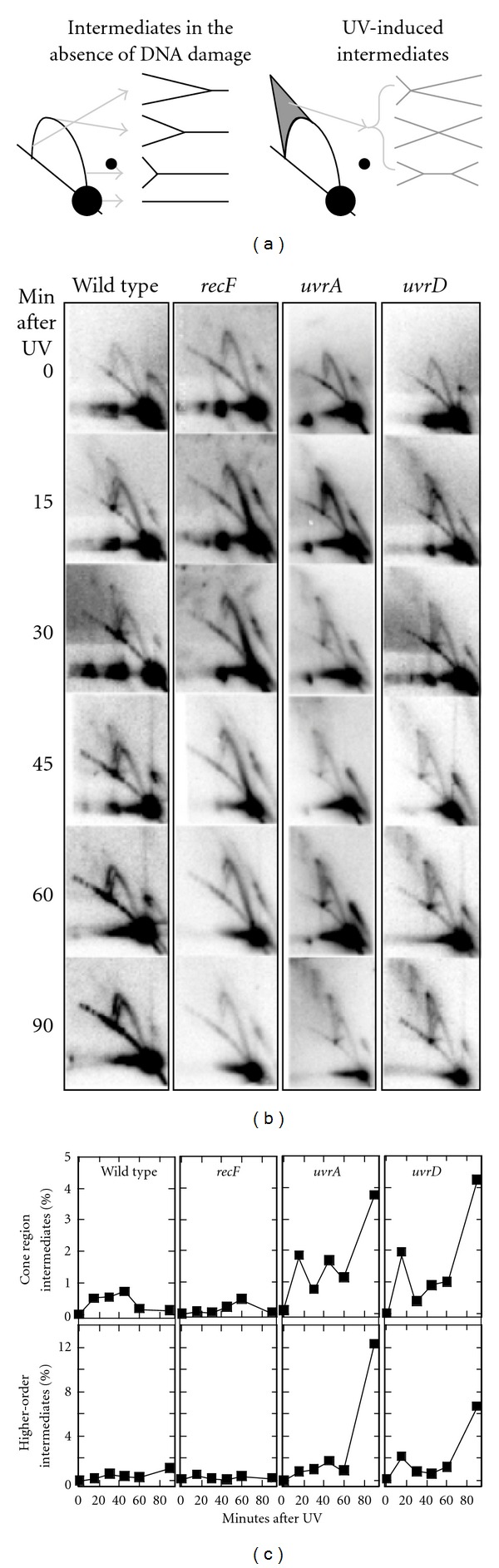
In the absence of UvrD, blocked replication forks persist leading to the accumulation of higher-order recombination intermediates in a manner similar to *uvrA *mutants. (a) Diagram of structural intermediates observed in the presence or absence of UV-induced damage. (b) Cells containing the pBR322 plasmid were UV-irradiated with 50 J/m^2^. At the times indicated, genomic DNA was purified, digested with PvuII, and the structural intermediates were examined by two-dimensional agarose gel analysis. Gels shown are representative of at least two independent experiments. (c) The percentage of UV-induced intermediates relative to nonreplicating plasmids over time is plotted. Percentages were quantified as the ratio of radioactivity in either the cone region or the high-order intermediate region over the amount of radioactivity in the nonreplicating region.

 Following the arrest of replication by UV-induced damage, the nascent DNA at the replication fork is displaced and partially degraded prior to the resumption of replication [4]. Recent studies have postulated that UvrD may function in clearing and processing of blocked replication forks, which may account for its failure to restore DNA synthesis [38, 44, 45]. Alternatively, UvrD may function at forks blocked by UV damage specifically in a nucleotide excision repair capacity. To determine which roles of UvrD may be required in replication recovery following UV damage, we examined whether UvrD contributes to the displacement and degradation of the nascent DNA at replication forks arrested by UV-induced damage. We reasoned that if the UvrD helicase were required to displace the nascent DNA, then the degradation of the nascent DNA at the arrested replication fork would be reduced in the protein's absence. To monitor DNA degradation, cultures grown in media containing ^14^C-thymine were pulse-labeled for 5 s with ^3^H-thymidine, collected on filters, resuspended in nonradioactive media, and immediately UV-irradiated. The amount of radioactivity remaining in the cultures was then followed over time. The dual radio-labeling allows us to simultaneously monitor the degradation that occurs in the total genomic DNA (^14^C) and the nascent DNA synthesized immediately prior to irradiation (^3^H). Following irradiation of wild-type cultures, the genomic DNA primarily remained intact and little or no degradation was detected (Figure 3). However, consistent with earlier studies, some limited degradation of the nascent DNA was detected at early times following irradiation [4, 8]. The loss of ^3^H-labeled DNA ceased at a time that correlated with the resumption of DNA synthesis and then began to increase (Figure 3). In principle, an increase in ^3^H should not be possible with this assay design. Previous work has shown that this increase is most likely due to remaining intracellular pools of radio-labeled thymidine that could not be washed away [5]. In the absence of RecF, which is required to limit the degradation at blocked replication forks, the nascent DNA degradation was more extensive and continued over a longer duration until approximately 50% of the nascent DNA has been degraded (Figure 3). In previous work, we have shown that the lagging strand is preferentially degraded following UV irradiation. This may explain why the degradation ceases after half of the nascent DNA has been degraded [4]. For the purposes of comparison, we also examined the degradation occurring in *recQ *and *uvrA *mutants. RecQ is a helicase that has been demonstrated to participate with RecJ to displace and degrade the nascent DNA at replication forks blocked by UV-induced damage [4]. In *recQ *mutants, no degradation of the nascent DNA was observed following irradiation and the remaining intracellular pools of ^3^H-labelled thymidine were rapidly incorporated (Figure 3). UvrA is required for the initial recognition step of nucleotide excision repair and is not thought to play any role in processing of the replication fork. Following irradiation of *uvrA* mutants, we observed that the nascent DNA degradation still occurred, consistent with what has been reported previously (Figure 3 and [4, 8]). When we examined UV-irradiated cultures of *uvrD*, we observed that degradation of the nascent DNA occurred and was similar in extent to that seen in *uvrA *mutants (Figure 3). The data indicate that when replication is arrested by UV-induced damage, UvrD is not required for and does not contribute to the degradation of the nascent DNA *in vivo*.

### 3.3. UV-Induced Replication Intermediates Accumulate and Persist in *uvrD *Mutants

 To further differentiate between a potential role for UvrD in nucleotide excision repair and in processing replication forks, we compared the structural intermediates that are formed at replication forks following UV irradiation in *uvrD *mutants to *uvrA *and *recF* mutants. Previous work has shown that defects in nucleotide excision repair or replication fork regression lead to different structural intermediates following arrest [6]. Intermediates were visualized on replicating molecules of the pBR322 plasmid using a two-dimensional (2D) gel electrophoresis technique. Replicating cells containing this plasmid were irradiated with 50 J/m^2^, a dose that produces approximately one lesion per plasmid strand [6]. Cells were harvested at various times after irradiation, and the DNA was purified and digested with PvuII, which linearizes the plasmid proximal to its unidirectional origin of replication. The replication intermediates were then examined using 2D agarose-gel electrophoresis and Southern analysis with ^32^P-labeled pBR322 as a probe. In the absence of damage, nonreplicating plasmids migrate as a linear 4.5 kb fragment, which forms the prominent large spot on the blot (Figure 4(a)). Replicating molecules, which form Y-shapes, migrate more slowly due to their increased size and nonlinear shape and appear as an arc extending out from the spot of linear plasmid fragments. Following irradiation of wild-type cultures, a transient cone region is observed above the arc of replicating Y-shaped molecules, consisting of X-shaped and double Y-shaped molecules (Figure 4). In previous work, we demonstrated that a portion of these molecules represent products that were formed by a RecF-catalyzed regression of the replication fork DNA [3, 6, 46]. These damage-induced intermediates begin to resolve after 30 min, at a time that correlates with the removal of lesions and the recovery of replication. In the absence of RecF, the arrested fork DNA is not maintained and these intermediate structures are not observed (Figure 4). By contrast, in *uvrA* mutants, the fork regression occurs normally but fails to resolve as the obstructing lesion is not removed from the DNA. In these mutants, the regressed fork intermediate is seen to persist and accumulate, forming higher-order, illegitimate intermediates by the end of the 90 min time course (Figures 4(b) and 4(c)).

 We reasoned that if UvrD was required to catalyze the regression of the fork DNA at UV-induced lesions, then the cone region intermediates would be reduced or absent in these mutants following UV irradiation. However, when we examined *uvrD *mutants, we observed elevated levels of these intermediates that accumulated throughout the 90 min time course (Figure 4). These intermediates went on to form the higher-order intermediates that are a hallmark of nucleotide excision repair-deficient mutants, consistent with the high levels of recombination and strand exchange seen in these mutants [9, 10].The presence of the fork regression products in *uvrD *mutants indicates that UvrD is not required to catalyze this reaction *in vivo*. Further, the similarity between the intermediates seen in *uvrA* and *uvrD* mutants would suggest that the failure of *uvrD *mutants to resume DNA synthesis after UV irradiation is most likely due to their inability to carry out nucleotide excision repair. 

## 4. Discussion

 In addition to its role in nucleotide excision repair, UvrD has also been postulated to catalyze fork regression and the displacement of the nascent lagging strand during the recovery of replication after arrest [36, 44, 45]. Here, we examined the functional roles for UvrD's contribution to cell survival and the recovery of replication following arrest by UV-induced damage. We observed that both the nascent strand processing and regression of the fork DNA occurs normally in the absence of UvrD. Rather than diminished levels of regressed fork intermediates forming in *uvrD* mutants, we observed that elevated levels of these intermediates formed and accumulated, similar to that seen in other nucleotide excision repair mutants. The observations are most consistent with the idea that the failure to restore replication in UvrD mutants is due to its role in nucleotide excision repair. 

 A role of UvrD in nucleotide excision repair, by itself, could sufficiently account for the hypersensitive and replication-defective phenotypes observed in *uvrD* mutants after UV irradiation. UvrD is required for the turnover of UvrC, which is not upregulated during the SOS response [47]. Thus, only a limited amount of repair occurs in the absence of UvrD, which is generally restricted to the repair of 6,4-photoproducts [40]. The minimal amount of nucleotide excision repair seen in *uvrD *mutants is consistent with it being modestly more resistant to UV damage than other repair mutants of this class. Otherwise, with respect to the processing of the nascent DNA, fork reversal, and impaired recovery of replication, *uvrD* mutants exhibit phenotypes nearly identical to those of other nucleotide excision repair mutants.

 The concept that UvrD may function in displacing the nascent DNA and promote fork reversal following arrest developed from a number of indirect genetic observations. A series of previous studies observed that in *recBC* mutants, which are defective in double-strand break repair, elevated levels of chromosome breaks can be detected in thermosensitive replication mutants, *dnaE *and *dnaN *(the catalytic subunit of Pol III and the Pol III clamp, resp. [48, 49]) at the restrictive temperature [37, 44, 45]. If cells were additionally mutated in *uvrD*, the level of detectable chromosome breaks was reduced. The authors speculated that these chromosome breaks arose as a result of replication forks collapsing to generate double-strand breaks. However, the assays employed in these studies were unable to address where the breaks form in the chromosome, and other studies have suggested that breaks repaired by RecBC do not form directly at the replication forks following arrest *in vivo* [4, 5, 50]. Curiously, these studies also noted that a different *uvrD *mutant lacking both ATPase and helicase activity failed to suppress chromosome breaks in these backgrounds. 

 When considering the differences between the results obtained in these studies, it is also important to consider the mechanism by which replication is arrested in each case. Whereas we used UV-induced damage to block the replication machinery, studies observing chromosomal breaks have often disrupted the replisome proteins themselves, using thermosensitive mutants. It seems probable that the biological events occurring after the loss of replication proteins would be distinct from those that occur when replication is blocked by impediments such as proteins or lesions, especially if one assumes that the replication proteins are required for the natural recovery process. Consistent with this, previous work from our lab has demonstrated a marked difference between the events following replication arrest caused by UV-induced damage and disruption of the DnaB helicase [51]. Whereas replication forks blocked by UV-induced lesions are protected and maintained by the RecFOR proteins, disruption or loss of DnaB helicase results in the collapse and degradation of the replication fork, a process that is antagonized by RecFOR function [51].

 Other genetic studies have inferred a role for UvrD in processing replication forks based on the synthetic lethality between *rep* and *uvrD* mutants [29, 35]. The Rep helicase is suggested to play a role in removing nucleoproteins, DNA secondary structures, or transcriptional machinery encountered by the replisome during replication [34, 52]. These observations have been interpreted to suggest that UvrD may be partially redundant with Rep function in removing nucleoprotein impediments encountered during replication [34]. However, both Rep and UvrD are directly associated with replication processes and it is unclear whether the synthetic lethality of *rep uvrD* double mutants can be attributed to the inability to overcome transcriptional blocks to replication or as a result of other impediments. 

 We have shown that when replication is blocked by UV-induced damage, it does not contribute to the displacement of the lagging strand or replication fork reversal but is required to carry out nucleotide excision repair before replication can resume. We do not rule out the possibility that UvrD contributes to fork processing when replication encounters other impediments, such as DNA-bound proteins, RNA polymerases, or even other forms of damage. It would be of interest to pursue these investigations in future studies as well as address how UvrD can generate chromosome breaks in the unusual case where replication proteins are targeted for disruption using thermosensitive mutants.
